# Color Changes and Mechanical Properties of Glass Fiber Reinforced Polycarbonate Composites after Thermal Aging

**DOI:** 10.3390/polym14020222

**Published:** 2022-01-06

**Authors:** Zhenbo Lan, Jiangang Deng, You Song, Zhuolin Xu, Yu Nie, Yanming Chen, Ye Ma

**Affiliations:** 1 Wuhan Nari Limited Liability Company of State Grid Electric Power Research Institute, Wuhan 430074, China; l17720559398@163.com (Z.L.); dengkelvin@163.com (J.D.); lan15926378682@163.com (Y.S.); xuzhuolinwh@139.com (Z.X.); whnr18327057305@163.com (Y.N.); 2 State Grid Electric Power Research Institute, Wuhan 430074, China; 3 School of Power and Mechanical Engineering, Wuhan University, Wuhan 430072, China; Chenyanming79@126.com; 4 Core Facility of Wuhan University, Wuhan University, Wuhan 430072, China

**Keywords:** glass fiber reinforced polycarbonate (GF-PC) composite, aging, chroma, mechanical properties

## Abstract

Thermal aging of polymer matrix composites exert significant influence on their properties and applications. This paper studied the color changes and mechanical properties of glass fiber reinforced polycarbonate (GF-PC) composites after aging at different temperatures, and the correlation between the trend of color changes and mechanical properties after aging was discussed. The GF-PC composites were aged at 85 °C, 100 °C, 115 °C, 130 °C and 145 °C, respectively. Thereafter, CIELAB colors were used to characterize the color changes of the composites after aging. Tensile and three-point bending tests were carried out to determine the mechanical properties of the composites. According to the values of CIELAB color, the color changes and the color difference (ΔE) of the GF-PC composites after aging were calculated, which showed that color of the GF-PC composite aged at 100 °C changed the most. The color changes of the composites after aging mainly comes from the change of brightness (L value), which was 25.067 for the Raw GF-PC composite. When the aging temperature increased from 85 °C to 100 °C, the brightness of the composites also increased, but decreased when the aging temperature is above 100 °C and continues to rise. Coincidentally, the trend of the mechanical properties of GF-PC composites is closely associated with color changes in the aging temperature range of 85 °C to 145 °C. The tensile and flexural strength of the composites reached the maximum value 72 MPa and 131 MPa, respectively, after aged at 100 °C. It can be speculated that the brightness of the GF-PC composites correlates with trends observed in its tensile strength and bending strength.

## 1. Introduction

Polymer matrix composites are widely used in mechatronics, manufacturing and chemical industries. As a kind of conventional reinforcement, glass fiber has good mechanical properties, fire resistance, corrosion resistance, low water absorption and good heat resistance. Researchers have found that glass fiber reinforcement can significantly improve the properties such as hardness, tensile strength, and flexural strength of polymer matrix composites [1,2,3,4,5,6]. However, the manufacturing, storage, and service conditions of glass fiber reinforced polymer matrix composites make them vulnerable to exposure to various environmental conditions, such as humidity, high temperature, oxygen, ultraviolet radiation and chemical media [7,8]. These factors will act on the polymer matrix through different interactive damage mechanisms, causing the destruction of the chemical structure, and resulting in a progressive deterioration of the physical, chemical and mechanical properties [7,8,9,10,11].

Researchers have conducted a lot of studies on properties of glass fiber reinforced polymer composites in different environmental conditions. Wang Meng et al. [12] explored the influence of high temperature environment on the thermal oxidative aging behavior and thermal degradation kinetics of brominated epoxy resin/Sb_2_O_3_ co-effective flame retardant short glass fiber reinforced nylon 10T composite. The results showed that the composite structure is seriously damaged and the thermal degradation behavior changes significantly. Han Yaozhang et al. [13] studied the influence of temperature, coupling agent content and thermal aging time on the bending and shear properties of glass fiber reinforced epoxy resin composites. The results showed that the bending strength decreased by 11.8% on increasing temperature. Gao Kun et al. [14] took epoxy resin/glass fiber composites as the research object, carried out wet and heat cycle aging test on them, and studied the changes of mechanical properties and dielectric properties of the composites as well as the mechanism of wet and heat aging. The results showed that the mechanical and dielectric properties of the composites decrease with the aging time. Wang Guojian et al. [15] studied the effect of high temperature aging on the properties of glass fiber reinforced epoxy vinyl ester resin matrix composites. The results showed that the tensile and bending properties of the composites increased at the early stage of aging but decreased gradually at the later stage. At 28 days of aging, the tensile and bending properties decreased by 6.14% and 9.23%, respectively. Mouzakis et al. [16] used quasi-static mechanics and dynamic mechanics to study the effects of temperature, humidity, ultraviolet radiation and their alternating circulation on the properties of composites. The results showed that the stiffness of composites increased after aging, but the strength decreased slightly.

Polycarbonate is a typical plastic material with density of 1.2 g/cm^3^, and glass transition temperature in the range 140–150 °C. Polycarbonate can replace some non-ferrous metals and steel materials, and finds application in glass assembly industry, automobile industry, electronics, electrical industry, and national defense industry. It has excellent impact resistance, transparency, fatigue resistance, dimensional stability, creep resistance, excellent electrical insulation, solvent erosion resistance, and wide operating temperature range (−130~130 °C) [17,18,19]. Therefore, it is necessary to focus on the influence of temperature on the microstructure and properties of polycarbonate [20,21,22]. For example, thermal aging is an inevitable process for polycarbonate at high temperature. Due to the presence of tertiary hydrogen atoms in the polymer chain, the polycarbonate molecule may produce carbon-carbon double bonds and deterioration as outcome of overheating. The color of the material gradually turns yellow and the transmittance decreases, meanwhile, surface defects such as cracks may develop gradually [9,23]. It is important to study the aging process of polycarbonate, understand the aging mechanism, and improve the anti-aging performance of polycarbonate and polycarbonate matrix composites.

This paper studied the color changes and mechanical properties of glass fiber reinforced polycarbonate (GF-PC) composites after aging at different temperatures. The correlation between the trend of color changes, microstructure and mechanical properties after aging was discussed, hoping to help understand the influence of aging on the properties of the composites and improve the aged properties of GF-PC composites.

## 2. Materials and Methods

### 2.1. Materials

**Figure 1:**
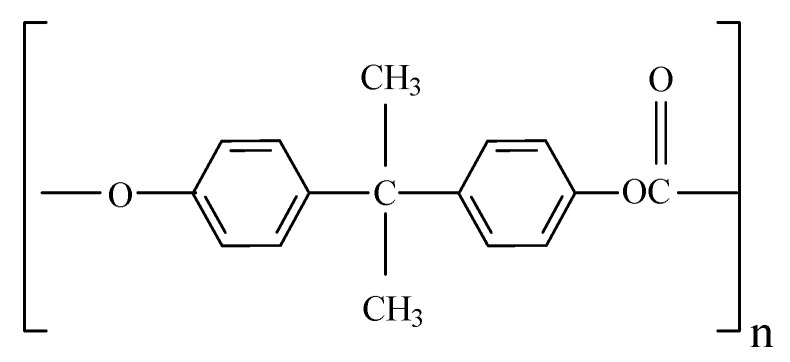
The formula chemical structure of the polycarbonate matrix 2.2 Thermal aging experiments.

GF-PC composites comprised of polycarbonate matrix and glass fiber reinforcement were prepared by the industrial pultrusion process. This method is a typical manufacturing process that can economically and continuously produce composite materials. The fiber is directly pultruded and enters the mold through the fiber distributor, then the extruder was used to inject the thermoplastic polycarbonate matrix melt into the mold. The fiber and thermoplastic polycarbonate matrix are immersed in the mold and then formed out of the mold. The formula chemical structure of the polycarbonate matrix is shown in Figure 1. The volume fraction of the glass fiber is about 40%.

According to ASTM D3045-92 [24] standard, thermal aging was performed using an oven (XMA-600) by heating the GF-PC composites at different temperatures (85 °C, 100 °C, 115 °C, 130 °C and 145 °C, respectively) for 8 h under air atmosphere.

### 2.2. Properties Tests

Color is represented by both brightness and chromaticity. Chromaticity is a property of color that does not include brightness. It reflects the hue and saturation of color. The CIELAB color [25] was used to assess the color of the composites. Accordingly, the color of composites was expressed in terms of 3 coordinate values (L, a, b), where L represents brightness ranging from 0 to 100, “a” represents red (0~+128) or green chromaticity (−128~0), and “b” is the color of yellow (0~+128) or blue chromaticity (−128~0). The CIELAB values of each specimen before and after aging at different temperatures were determined with a Minolta CR-321 colorimeter. The Minolta CR-321 colorimeter uses a test light source with 400–700 nm wavelength range, circumferential 10-degree illumination, and 0 degree viewing angle geometry to measure a circular area with a diameter of 3 mm. Mean CIELAB values were determined after 10 separate measurements in succession, repositioning the specimen after each measurement. The same examiner made all colorimetric measurements.

The chromatic aberration (ΔL, Δa, and Δb) of the GF-PC composites after aging can be achieved by equations as follow:

ΔL = L_A_ − L_B_ (1)

Δa = a_A_ − a_B_ (2)

Δb = b_A_ − b_B_ (3)

where the subscript B means before aged and A means after aging. The CIELAB values of raw PC are taken as the color of the composite before aging. Furthermore, the color stability of the composites can be assessed by determining the color difference (ΔE) between CIELAB values before and after aging. Mean ΔE values were calculated for different aging temperatures using the following formula:

ΔE = [ΔL^2^ + Δa^2^ +Δb^2^]^½^ (4)

The tensile and bending tests of the aged GF-PC composites were carried out at room temperature by using an MTS E45 loading frame (MTS, Shenzhen, China). The gauge length of the tensile samples are 65 mm, and the width and thickness are 10 mm and 4 mm, respectively. The sample size of the three-point bending test is 10 × 4 mm^2^, and the span of the bending test is 15 mm.

Scanning electron microscope (SEM, TESCAN MIRA 3 LMH, Brno, Czech Republic) with an acceleration voltage of 5 kV was used to observe the tensile fracture morphology of raw and aged GF-PC composites. Since the polycarbonate matrix is an electrical insulator, a layer of platinum was deposited on the surface before observation.

## 3. Results

Means of CIELAB values for each specimen are listed in Table 1. According to the data in Table 1, the CIELAB values of the raw GF-PC composite before aging was about L = 25.067, a = −0.556, and b = −0.462, respectively. After aging, the color of the composites changed with the aging temperature. The value of “L” shows a trend of increasing first and then decreasing, and reaches the peak value of 27.077 after aging at 100 °C, while the negative value of “a” keeps increasing slowly and “b” remains almost unchanged. The increasing trend of “L” indicates that the brightness of the composite is going up with the increase of aging temperature. However, when it is above 100 °C, the brightness of the composites declined to 24.475 for 145 °C aged sample, which is even lower than that of the raw GF-PC composite. The negative value of “a” is indicative of the slightly green chromaticity of the composites, and its small rise indicates that the composites are becoming less green. The small fluctuation of “b” value indicates that the blue colors of the composites are almost unchanged, but the negative value of b decreases obviously after 145 °C aging, indicating that the blue color of the 145 °C aged composite is significantly increased.

The calculated chromatic aberration (∆L, ∆a, and ∆b) and color difference (∆E) are listed in Table 2. ∆E of the composites is the largest after aging at 100 °C, which mainly comes from the increase of the brightness of the composites. It is clear that “L” changes the most for any aging temperature, and small changes in “a” and “b” hardly affect the results of ∆E.

**Figure 2:**
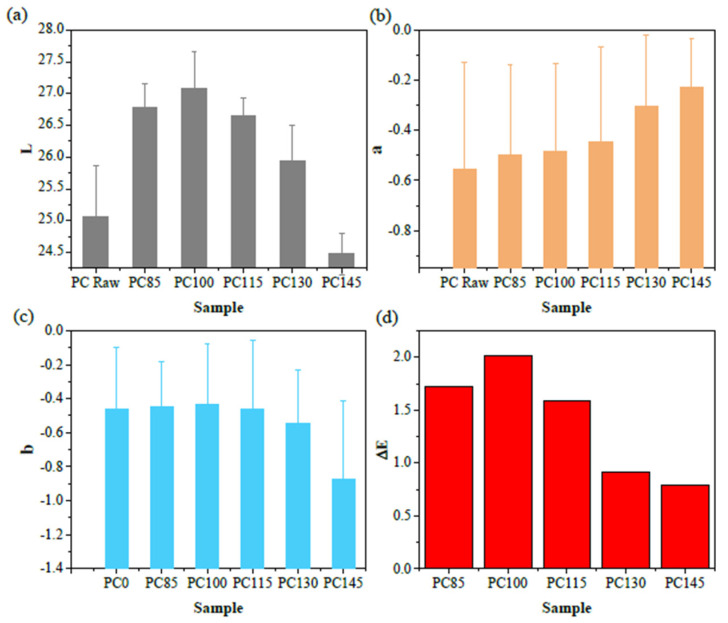
The CIELAB values and ΔE of the GF-PC composites before and after aging. (**a**) “L” of the composites; (**b**) “a” of the composites; (**c**) “b” of the composites; (**d**) “ΔE” of the composites after aging.

Figure 2 displays the CIELAB values and ΔE of the GF-PC composites before and after aging. As can be seen from Figure 2d, the variation trend of ΔE is very similar to that of ΔL, which show a trend of rising first and then falling, reaching the maximum for 100 °C aged sample. Therefore, the color of GF-PC composites especially brightness changed the most under the aging condition of 100 °C.

**Figure 3:**
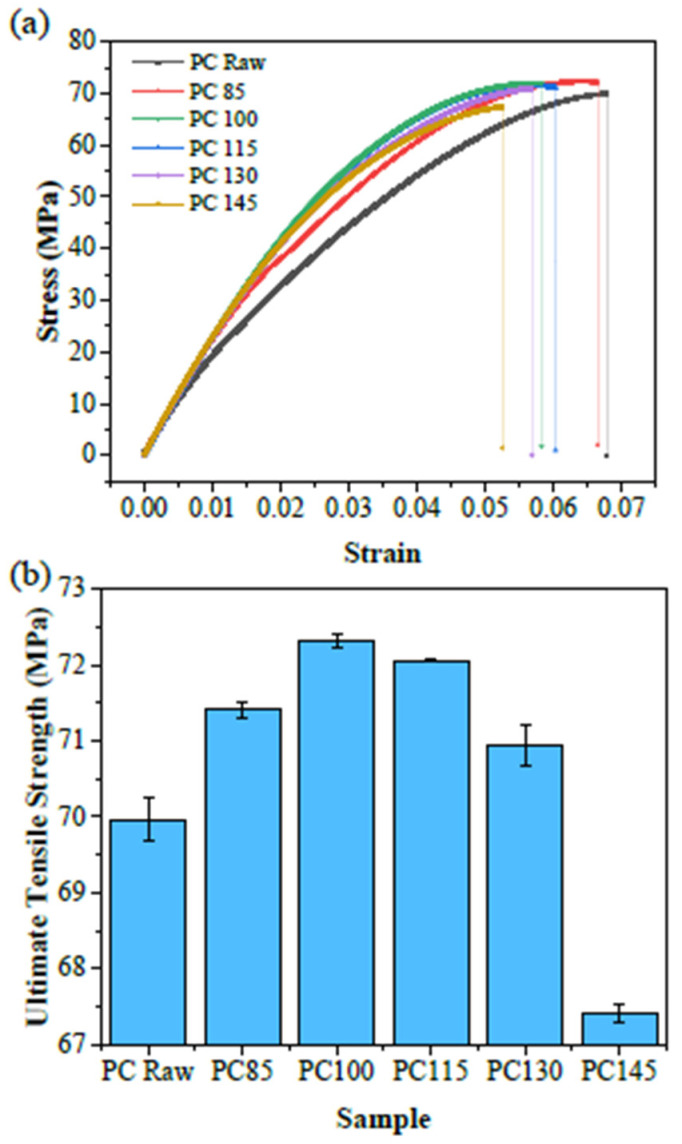
Tensile properties (**a**) The tensile stress-strain curves of the GF-PC composites before and after aging; (**b**) The ultimate tensile strength of the GF-PC composites before and after aging.

The tensile properties of the GF-PC composites are displayed in Figure 3. The tensile stress-strain curves in Figure 3a show that the modulus of the composites after aging increases compared with the raw material. This fact may be explained by the material densification and crosslinking of glass fibers occur easily at high temperature, the adhesion at the fiber/polymer interface is also stronger under high temperature conditions, which probably result in an increase in the strength of the GF-PC composites [20,26,27]. Figure 3b displays the ultimate tensile strength (UTS) of the GF-PC composites. The trend of UTS showed a slight increase first and then decrease as the aging temperature increased. It reaches to the maximum of 72 MPa after aging at 100 °C, and then decreased at higher aging temperatures. Moreover, the UTS tendency is consistent with the observation in color changes (Figure 2d). The trend of UTS of the aged GF-PC composites is very similar to that of ΔE or even “L”. After aging at 100 °C for 8 h, the brightness (L value) of the GF-PC composite significantly increased, ΔE rose to the maximum of 2.013, while the UTS of the composite also attained it highest value of 72 MPa. However, when aged at higher temperatures, the lightness of the composites declined rapidly. ΔE, which is significantly affected by the change in L, also showed a sharp downward trend. In addition, the tensile strength for GF-PC composites increases after aging at 85 °C and 100 °C, respectively, which can be attributed to the fact that the oxygen concentration on the surface of PC is much higher than that on the inside. Therefore, oxidation reactions occur on the surface first and form a thin layer of oxidation products which can harden the material and increase the tensile strength [28]. On the contrary, after aging at the higher temperature of 145 °C at which the polycarbonate matrix is very close to the rubbery state, the tensile strength of the GF-PC composite decreased a lot. Richeton et al. [29] found that the yield stress and the strain hardening of PC decreased as the temperature was increased. This phenomenon might be due to the volatilization of small molecules produced by oxidation which leaves defects on the surface, local stress concentration sites formed after being stressed, thereby resulting in the decline of the UTS [28,30].

**Figure 4:**
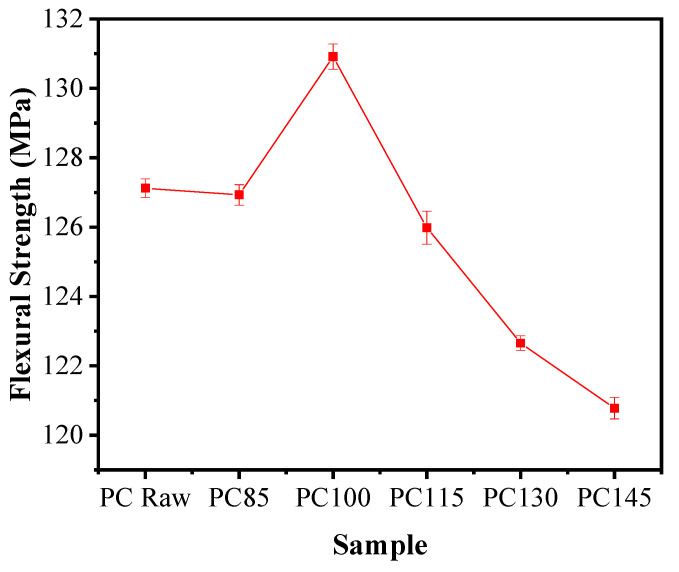
The flexural strength of the GF-PC composites before and after aging.

Figure 4 showed the flexural strength of the GF-PC composites before and after aging. Consistent with the trend of tensile strength, the flexural strength of the composites increased after aging at 100 °C, and then declined with the increasing aging temperature. When aged at lower temperature such as 85 °C and 100 °C, PC matrix crystallization is improved rapidly in the aging temperature environment, thus leading to an increase of mechanical strength. The increase of crystallinity plays a dominant role which results in an enhanced flexural strength, however, the degradation caused by aging declines the mechanical properties [20,26,27]. At higher aging temperature, PC matrix crystallization is basically perfect, degradation caused by aging is the main factor causing the change of mechanical properties. At this time, a large number of highly active free radicals and peroxides accumulated in PC matrix caused the rapid reduction of its flexural strength [28,29,30].

**Figure 5:**
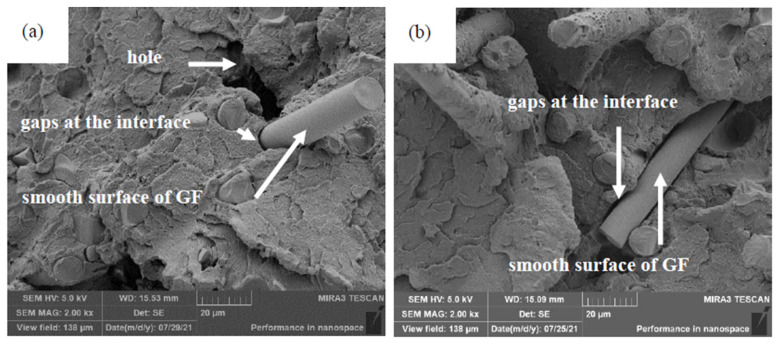

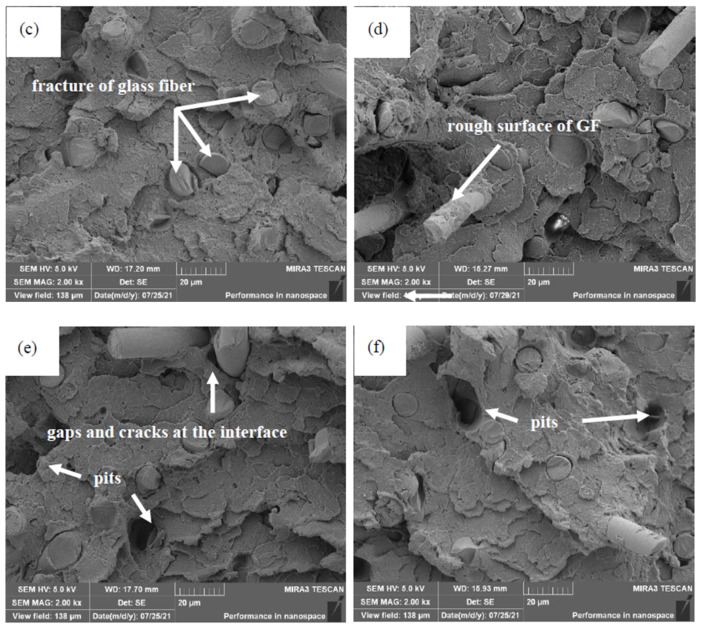
Morphology of tensile fracture of the GF-PC composites. (**a**) Raw material; (**b**) Aged at 85 °C; (**c**) Aged at 100 °C; (**d**) Aged at 115 °C; (**e**) Aged at 130 °C; (**f**) Aged at 145 °C.

Figure 5 shows the morphology of the tensile fractures of the composites. It is clear that the glass fiber embedded in the PC matrix. On tensile fractures, some glass fibers break, while some pull out of the matrix and leave many holes, indicates that the glass fiber reinforced the PC matrix. As can be seen from Figure 5a, the surfaces of the glass fibers are very smooth in the cross section of the GF-PC composite before aged. Some fibers were detached from the PC matrix and left holes on fracture during the tensile process. After aging at 85 °C (Figure 5b), the PC on the fiber surface degraded and began to fall off. When the composite was aging at 100 °C (Figure 5c), the glass fibers are closely covered by PC matrix. Most of the glass fibers break on fracture, and it is rare to see the glass fibers detach and pull out of the matrix, indicating that the PC resin is well bonded with the fiber. As the aging temperature increased (Figure 5d–f), the resin on the sample surface degraded and fell off, and the fibers exposed to the matrix surface were very clear. A large number of pits appeared on the matrix surface, gaps and cracks appeared on the interface between the fibers and the polycarbonate matrix. These phenomena are attributed to the difference in the coefficient of thermal expansion (CTE) between the glass fiber and the polycarbonate matrix. During the thermal aging, micro-residual stresses arise from the differential coefficient of thermal expansion of the glass fiber and polycarbonate matrix, which result in interface damage and cracks on the surface between the fiber and the matrix [31,32]. These generated residual stresses promote the onset of cracking and delamination in the fibers of the composites.

## 4. Conclusions

The color changes and the mechanical properties of the GF-PC composites after aging at different temperatures were investigated. Within the limitations of this study, the following conclusions can be drawn:

1. Comparing with the raw GF-PC composite, the color of the composites after aging at high temperatures changed obviously. The color difference (ΔE) is mainly reflected in brightness of the composites, which increased for 85 °C and 100 °C, but decreased after aging at high temperature.
2. The tensile and flexural strength of the composites reached the maximum value of 72 MPa and 131 MPa, respectively, after aging at 100 °C for 8 h, which is of guiding significance to improve the mechanical properties of industrial GF-PC composites.
3. In the aging temperature range of 85 °C to 145 °C, the mechanical properties are closely related to the color difference of the GF-PC composites. It can be inferred that the trend of changes in the brightness of the composites is consistent with trends of changes observed in its tensile strength and flexural strength.

## Figures and Tables

**Table 1:** The CIELAB values of the GF-PC composites before and after aged.

Sample	L	a	b
PC Raw	25.067	−0.556	−0.462
PC85	26.789	−0.500	−0.449
PC100	27.077	−0.483	−0.434
PC115	26.647	−0.444	−0.463
PC130	25.945	−0.303	−0.545
PC145	24.475	−0.227	−0.875

**Table 2:** The chromatic aberration and ΔE values of the GF-PC composites after aged.

Sample	ΔL	Δa	Δb	ΔE
PC85	1.722	0.056	0.013	1.723
PC100	2.010	0.073	0.028	2.013
PC115	1.580	0.112	−0.001	1.584
PC130	0.878	0.253	−0.083	0.917
PC145	−0.592	0.329	−0.413	0.793
